# Optimizing Tobacco-Free Workplace Programs: Applying Rapid Qualitative Analysis to Adapt Interventions for Texas Healthcare Centers Serving Rural and Medically Underserved Patients

**DOI:** 10.3390/cancers17152442

**Published:** 2025-07-23

**Authors:** Hannah Wani, Maggie Britton, Tzuan A. Chen, Ammar D. Siddiqi, Asfand B. Moosa, Teresa Williams, Kathleen Casey, Lorraine R. Reitzel, Isabel Martinez Leal

**Affiliations:** 1 School of Medicine, Baylor College of Medicine, One Baylor Plaza, Houston, TX 77030, USA; hannah.wani@bcm.edu; 2 Department of Behavioral Science, The University of Texas MD Anderson Cancer Center, 1155 Pressler Street, Houston, TX 77030, USA; mbritton@mdanderson.org (M.B.); abmoosa@mdanderson.org (A.B.M.); lreitzel@mdanderson.org (L.R.R.); 3 Department of Psychological, Health & Learning Sciences, The University of Houston, 3657 Cullen Blvd., Stephen Power Farish Hall, Houston, TX 77204, USA; tchen3@central.uh.edu; 4 HEALTH Research Institute, The University of Houston, 4349 Martin Luther King Blvd., Houston, TX 77204, USA; 5 Department of Management, Policy, and Community Health, School of Public Health, University of Texas Health Science Center at Houston, 1200 Pressler St., Houston, TX 77030, USA; 6 Integral Care, 1430 Collier St., Austin, TX 78704, USA; teresa.williams@integralcare.org (T.W.); kathleen.casey@integralcare.org (K.C.)

**Keywords:** cancer prevention, healthcare disparities, tobacco cessation interventions, tobacco-use disparities, medically underserved, social determinants of health, implementation science, rapid qualitative analysis, tobacco-free workplace policies, rural tobacco use

## Abstract

Background: Tobacco use is disproportionately high in rural areas, contributing to elevated cancer mortality, yet it often goes untreated due to limited access to care, high poverty and uninsured rates, and co-occurring substance use disorders (SUDs). This study explored the utility of using rapid qualitative analysis (RQA) to guide the adaptation of a tobacco-free workplace program (TFWP) in Texas healthcare centers serving adults with SUDs in medically underserved areas. Methods: From September–December 2023 and May–July 2024, we conducted 11 pre-implementation, virtual semi-structured group interviews focused on adapting the TFWP to local contexts (N = 69); 7 with providers (*n* = 34) and managers (*n* = 12) and 4 with patients (*n* = 23) in 6 healthcare centers. Two qualified analysts independently summarized transcripts, using RQA templates of key domains drawn from interview guides to summarize and organize data in matrices, enabling systematic comparison. Results: The main themes identified were minimal organizational tobacco cessation support and practices, and attitudinal barriers, as follows: (1) the need for program materials tailored to local populations; (2) limited tobacco cessation practices and partial policies—staff requested guidance on enhancing tobacco screenings and cessation delivery, and integrating new interventions; (3) contradictory views on treating tobacco use that can inhibit implementation (e.g., wanting to quit yet anxious that quitting would cause SUD relapse); and (4) inadequate environmental supports—staff requested treating tobacco-use training, patients group cessation counseling; both requested nicotine replacement therapy. Conclusions: RQA identified key areas requiring capacity development through participants’ willingness to adopt the following adaptations: program content (e.g., trainings and tailored educational materials), delivery methods/systems (e.g., adopting additional tobacco care interventions) and implementation strategies (e.g., integrating tobacco cessation practices into routine care) critical to optimizing TFWP fit and implementation. The study findings can inform timely formative evaluation processes to design and tailor similar intervention efforts by addressing site-specific needs and implementation barriers to enhance program uptake.

## Simple Summary

Despite smoking rates of 65–87%, adults with substance use disorders are rarely treated for tobacco use in healthcare centers when receiving treatment for non-nicotine substance use disorders in rural and medically underserved areas. Translation of evidence-based interventions for tobacco control is urgently needed to address tobacco-related cancer disparities among this group. We use a rapid qualitative analysis approach to advance adaptation of a tobacco-free workplace program in Texas healthcare centers serving adults with substance use disorders in rural and medically underserved areas. Rapid analysis of separate group interviews with staff and patients produced actionable findings to quickly optimize program fit, better address real-world conditions and program barriers, and enhance implementation to local contexts. This cost-effective, collaborative approach provides a framework for researchers to address site-specific needs in close to real-time to implement tobacco cessation efforts that reduce tobacco-related cancers for these disparately impacted patients in rural areas.

## 1. Introduction

Tobacco use remains the leading cause of morbidity and mortality in the United States (US) [1] and is causally linked to 17 different types of cancer, cardiovascular disease, chronic obstructive lung diseases, and stroke [2]. While overall smoking rates have declined nationally, smoking prevalence among individuals with substance use disorders (SUDs) remains disproportionately high, ranging from 65% to 87% depending upon the specific diagnosis [3]. These individuals tend to smoke more heavily and are more nicotine-dependent, with fewer successful quit attempts [4,5]. Accordingly, tobacco use causes more deaths among individuals with SUDs than the SUD for which they receive treatment [5], with over 53% of such individuals dying of tobacco-related causes and accounting for up to 50% of annual smoking-related premature deaths [6]. Additionally, within rural and medically underserved areas, commonly low socioeconomic status regions, tobacco-use rates are greater, and SUDs are frequently under-diagnosed and under-treated [7]. One US-based study found that only 10.6% of individuals with SUDs received treatment for their tobacco addiction [8]. Healthcare centers in rural and medically underserved areas often lack the infrastructure and resources for tobacco-use education, prevention, cessation, and treatment [9]. Limited access to transportation, lower income and insurance coverage, and limited access to medical services for cessation and treatment compound these challenges [10]. Beyond limited financial abilities and access to resources, individuals living in rural and medically underserved areas also struggle with inadequate access to care providers; thus, they are less likely to receive recommended prevention protocols and intervention opportunities [10]. As a result, individuals in these settings are less likely to receive tobacco-related interventions, contributing to higher cancer incidence and mortality [11]. Consequently, there is a great need to address tobacco addiction within this priority group of individuals with SUDs living in rural and medically underserved areas.

To reach these groups effectively, it is essential to engage existing care systems, such as substance use treatment centers (SUTCs) and medical healthcare centers (MHCs), including Federally Qualified Health Centers (FQHCs) [12,13]. FQHCs are community organizations that receive state and federal benefits to provide comprehensive primary and preventive care to medically underserved communities [14]. High tobacco-use rates (30%) have been reported among patients seeking SUDs care at FQHCs [15]. A recent study in such settings found that 83% of patients expressed a desire to quit tobacco, highlighting a missed opportunity, given the potential for tobacco cessation support to be embraced by patients [16]. However, individuals seeking treatment within MHCs and SUTCs for non-nicotine SUDs are not often offered tobacco cessation services; a 2023 national study indicated that within Texas, 76.6% of SUTCs screened for tobacco use, 68.3% offered tobacco cessation counseling, 42.6% offered nicotine replacement therapy (NRT), and 42.9% offered non-nicotine tobacco cessation medications [17]. Only 68.9% were found to have tobacco-free workplace (TFW) policies, which prohibit any tobacco use (cigarettes, e-cigarettes, chew tobacco, etc.) within company property, including indoor and outdoor areas, parking lots, and company vehicles [17]. Moreover, many centers only partially or inconsistently adopt tobacco control initiatives, hindering their effectiveness [17,18], leaving much work to be done to better align with best practices in tobacco control and intervention [13,19]. Thus, there is a necessity to bridge the gap in implementing evidence-based tobacco cessation interventions at SUTCs and MHCs to address the disproportionate burden of tobacco use.

Evidence-based TFW programs are effective in improving quit rates in SUTCs [20,21,22,23,24,25,26], yet implementation remains limited within these practice settings [13]. Several factors contribute to this gap, including lack of provider tobacco intervention education and training, ubiquity of on-site tobacco use, and undervaluing of tobacco dependence as a serious addiction. Although empirical evidence has shown that concurrent, rather than discrete, treatment of tobacco and substance use is more effective and can prevent SUD relapse by 25%, SUTC providers mistakenly perceive that patients are disinterested in tobacco cessation and that addressing tobacco use will compromise recovery from non-nicotine substances [13]. Providers also lack specialized training to effectively treat dependence and have limited capacity due to competing demands, preventing attention to tobacco cessation [27,28]. There is limited research available on the processes influencing implementation of tobacco cessation care within MHCs and SUTCs in rural and medically underserved areas. This evident gap in the translation of evidence-based practices for tobacco management within these settings warrants further investigation to understand the existing barriers and address high patient tobacco use among these priority populations.

Traditional qualitative approaches are a valuable tool in implementation science, integral to understanding the contextual factors impacting implementation [29]. However, such methods are time-consuming and resource-intensive, presenting a significant challenge in studies where real-time data is needed to inform the implementation process [30]. Rapid qualitative analysis (RQA) offers a streamlined alternative that maintains scientific rigor while enabling more timely feedback for program development and implementation [30]. The main benefits of RQA include reduced cost and time, increased amount of data collected, improved accuracy and efficiency, and the generation of a more accurate representation of participants’ realities [31]. Compared to traditional approaches, RQA prioritizes practical insight over extensive coding and focuses on identifying key mechanisms, intervention factors, and facilitators and barriers [32]. The use of rapid analysis methods allows key stakeholders and decision makers to develop timely solutions and inform program implementation more quickly without compromising rigor [32].

This study fills a gap in the literature regarding the use of rapid techniques in qualitative research to facilitate successful implementation of tobacco cessation interventions aimed at reducing tobacco-related cancers within rural and medically underserved settings. The aim of this study is to examine how the findings from RQA methods informed the adaptation and implementation of a comprehensive tobacco-free workplace program to meet the particular needs of SUTCs and MHCs serving adults with SUDs.

## 2. Materials and Methods

### 2.1. Intervention: Taking Rural Texas Tobacco Free

Taking Texas Tobacco Free (TTTF) is a comprehensive TFW program that partners with community and healthcare organizations in Texas to build capacity for sustained tobacco-use management. The aim of the intervention is to transform organizational culture regarding treating tobacco dependence through changing tobacco-use norms and ultimately reduce tobacco-related cancers. TTTF relies on evidence-based strategies to reduce tobacco-related inequities and stigmatization among those with the highest tobacco-use rates—marginalized and minoritized groups (e.g., those with substance use and/or mental health disorders, who are under-resourced, experiencing homelessness, are being treated for opioid use, or identify as members of sexual and gender minorities). To date TTTF has been funded through 11 awards to work with various community and healthcare centers serving these priority groups disparately impacted by tobacco use through targeted development and adaptation of the program to the diverse needs and characteristics of these distinct populations [19,20,21]. This study describes the Taking Rural Texas Tobacco Free program, focused on addressing tobacco dependence among healthcare patients with SUDs within rural and medically underserved areas in Texas [33].

The program uses a formative evaluation process as part of a mixed methods approach to adapt interventions to the needs and characteristics of the particular populations being served to enhance implementation. As program implementation is driven by a process of behavior change, TTTF focuses on transforming organizational culture to change the behavior of healthcare professionals in treating tobacco dependence who then affect patients’ tobacco-use behaviors. The mechanisms underpinning the change in organizational culture TTTF seeks to engender are informed by different theories, including Social Cognitive Theory that focuses on changing the behavior of healthcare professionals through shifting norms around treating tobacco use [34]. The Ecological Framework also guides program implementation as organizational capacity to affect change is built through training of staff and providers to address tobacco dependence within various healthcare settings through targeting implementation barriers across multiple levels—organizational, provider, and patient [35]. Additionally, the Theory of Organizational Readiness is used to guide program implementation through identifying organizational priority issues and needs to facilitate adaptation [36].

TTTF utilizes a Hybrid Type III design that involves assessing both the implementation of an intervention as well as its clinical effectiveness and outcomes [37]. This multi-component program is evidence-based and includes (1) the implementation and enforcement of an organization-wide TFW policy that includes all tobacco products (combustible, vaping, and chew and smokeless tobacco); (2) specialized staff education and training on assessing and treating tobacco dependence; and (3) integration of tobacco use assessments and delivery of evidence-based cessation services (e.g., brief interventions, tobacco cessation counseling, and NRT distribution) into routine practice for patients and staff. TTTF uses a host of recognized implementation strategies to change healthcare practice, including providing program partners with ongoing consultation and practical, hands-on-guidance throughout implementation as well as treatment resources (i.e., an NRT starter kit, tobacco-free signage and dissemination and educational materials) at no cost to them [38]. Active implementation for program partners followed a suggested blueprint and timeline.

### 2.2. Study Design, Recruitment, and Participants

This study reports on the findings from the qualitative component of a mixed methods project, focusing on the formative evaluation phase used to adapt and appraise implementation of a comprehensive TFW program within healthcare settings in medically underserved and rural areas. The definition of formative evaluation used here is that proposed by Stetler (2006) as “a rigorous assessment process designed to identify potential and actual influences on the progress and effectiveness of implementation efforts” [39]. Formative evaluation can occur before, during, or after implementation to maximize fit, uptake, and program success, and can be characterized according to the four progressive stages of development/diagnosis, implementation, progress, and interpretation. The current study utilized developmental formative evaluation processes in which pre-implementation interview data were collected to adapt the intervention to each setting.

**Figure 1:**
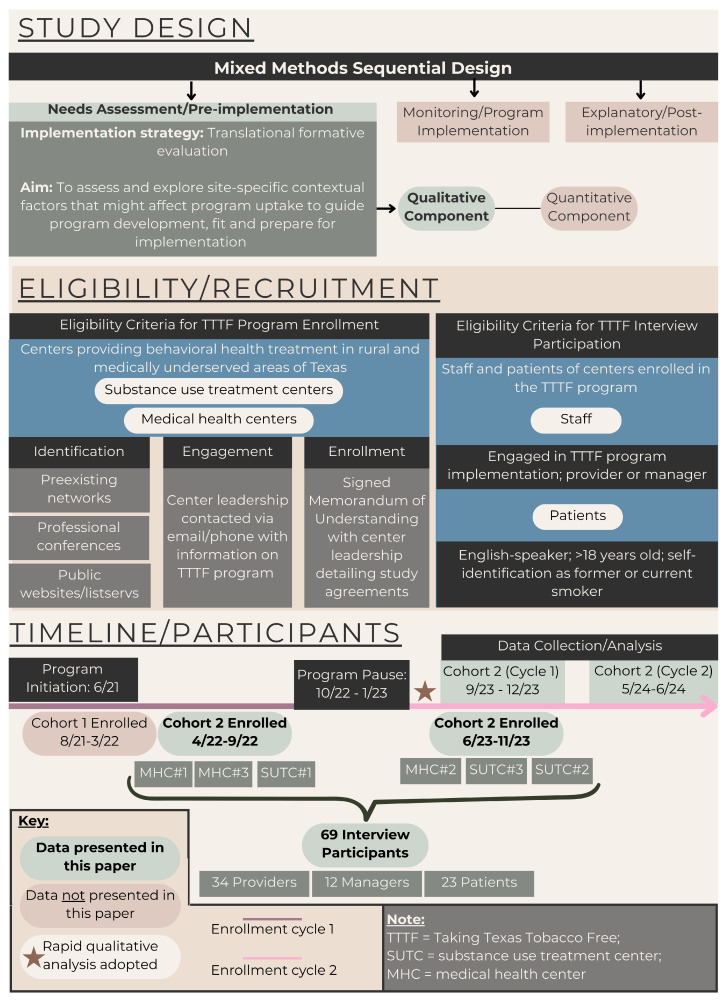
Study design, eligibility and recruitment, and timeline and participants.

Potential partners were recruited for participation in a multi-year funded project through continuing cycles of enrollment. For purposes of this study, enrolled centers were organized into cohorts to facilitate description of their enrollment timeframe and the RQA process. Details on the study design, recruitment process, eligibility and types of participants, and project timeline are detailed in Figure 1. The current study reports on the 2nd recruitment cycle, or cohort of program partners that included 6 centers (Table 1). The 1st cohort of program partners was excluded from the present study as the research team adopted rapid qualitative analysis—of which the effects upon program adaptation and implementation is the focus of this work—following recruitment of the 2nd cohort. Half of the 6 centers were recruited between June 2023–November 2023, and 3 initially recruited between April 2022–September 2022. However, in October 2022, the research team transferred to a different academic institution which resulted in a pause of ~3 months of research activities while regulatory and financial processes were established at the new institution. Our program partners were notified of this transfer of institutions and that research activities would be temporarily paused. To accommodate this hiatus in the grant timeline, the implementation team reached out to re-engage these 3 centers in the program, and the active implementation period for these participating centers was extended from generally 12 to 16 months.

A total of 11 virtual group interviews (N = 69) were conducted; 7 with staff who were providers (*n* = 34) and managers (*n* = 12), as well as 4 with patients (*n* = 23) from participating centers. Hereafter, staff is understood to include providers, managers, and other center employees who directly interacted with patients, unless otherwise specified. Although all program partners were solicited for permission to interview patients, many declined participation either out of a desire to protect their patients or because scheduling virtual group interviews was not feasible. Our aim was to understand staffs’ and patients’ perceptions, experiences, and needs around treating tobacco dependence, and to identify factors enabling or hindering implementation of TTTF to inform the tailoring of this tobacco-free workplace program to their settings. A common purposeful sampling strategy, criterion sampling, which consists of selecting participants based on predetermined criteria, was used to recruit staff and patients [44]. Recruitment of staff and patient group interview participants was coordinated through the center program champion—a volunteer manager or provider who was sponsored by TTTF to attend a 5-day Tobacco Treatment Training to become a tobacco treatment specialist, and who did not receive additional financial compensation for this position [45].

### 2.3. Institutional Approvals

This project was approved by the Internal Review Board (IRB) of the University of Houston (STUDY00002885, initial approval 20 April 2021). In October 2022, all research procedures were paused prior to the research team transferring to the University of Texas MD Anderson Cancer Center (MDACC), pending regulatory approvals at the new institution. MDACC determined that as the TTTF project is focused on quality improvement in healthcare it does not meet the regulatory definition of human subjects research and required Quality Improvement Assessment Board (QIAB) approval. The QIAB at MDACC approved study procedures (no study ID, initial approval 21 November 2022). While the QIAB seeks to optimize potential patient benefits from research and assures their safety, it is not an ethics committee like an IRB that focuses on ethical considerations and human subjects protection in research. However, as noted in the following Section 2.3, best research practices were applied for qualitative procedures, including informed consent processes and confidentiality and privacy procedures, and all participants provided oral consent prior to study participation. The difference in type of regulatory approval is based on the determinations of the different academic institutions.

### 2.4. Data Collection

Group interviews were carried out prior to implementation of program components, with the exception of a 90 min tobacco education training that was delivered to all staff at partnering centers. The rationale for this exception is that this training also served to educate and familiarize staff on the components of the TTTF program prior to the group interviews. Additionally, based on availability of trainings, and accommodations to our regular timeline in response to our program pause, some program partners received the 5-day specialized Tobacco Treatment Training as part of the process of preparing them to oversee program implementation, prior to the group interview. Separate semi-structured interview guides were used to conduct pre-implementation virtual group interviews with staff and separately with patients between September 2023–June 2024. Group interviews lasting from 40–60 min were conducted in-person or virtually and recorded using Zoom, a videoconferencing platform, by a cultural anthropologist and public health researcher (IML) trained in qualitative research. Interviews with staff and patients consisted of 4–10 participants in each group. The study specifics and voluntary nature of interview participation—i.e., right to decline to answer questions or to withdraw from the study without penalty—were discussed with participants who each provided oral consent prior to study involvement and/or being audio/video-recorded. Participants were also assured that their personal identifiers would not be retained, and only de-identified data would be used. The research aims as well as prior research experience guided the development of the different interview guides for staff and patients, which were field-tested and revised based on participants’ responses. Staff pre-implementation interview questions included a review of program materials—educational brochures, programs, and posters—to elicit participants’ feedback and cooperation in tailoring or creating materials to fit individual centers’ populations (i.e., in accordance with gender, race, ethnicity, language, sexual orientation, and age); specific types of tobacco-related services currently provided; tobacco assessment and supports offered; existing TFW policies; prior tobacco treatment education or training; anticipated barriers and facilitators to implementation of tobacco cessation interventions; program compatibility with existing workflows and practices; individual concerns regarding program implementation; leadership support for treating tobacco dependence; and any unique center needs that might impact program implementation. Patient pre-implementation questions focused on personal experience with tobacco use and quitting; perceptions of organizational and staff attitudes towards tobacco use; center-provided services to quit tobacco use; supports/strategies that had been most effective to quit tobacco; prior knowledge/education on the harms of tobacco use; and attitudes and concerns regarding implementation of a tobacco-free workplace program within the center. These pre-implementation interview questions sought to understand the individual context and needs of each center regarding their organizational characteristics and culture, the populations they served, attitudes towards addressing tobacco dependence within their particular setting, and expected program challenges or supports regarding implementation. Staff and patients were compensated for interview participation; staff received a $20, and patients, a $25 e-gift card. Additionally, via a baseline leadership survey, organizational and patient characteristics of participating centers were collected that focused on demographics, current tobacco-free policies and procedures, and organizational readiness of program partners.

### 2.5. Data Analysis

Data collection and analysis were conducted over 2 different phases lasting ~3 months, between September and December 2023, and May and June 2024, based on continuous recruitment cycles of participating centers. Enrolled centers were organized into cohorts for the purposes of this study to describe the timeframes of their enrollment and the RQA process. All interviews were audio-recorded and transcribed verbatim by a professional transcription service and checked for accuracy. Data were analyzed using a rapid analytic approach that included the use of structured templates and matrices [46]. This RQA method was developed specifically for use in health services and implementation research which often require a quicker turnaround time for qualitative data to inform program implementation and/or subsequent data collection [47]. Summary templates were developed for each of the different interview guides, organized according to domains or key concepts that corresponded with each interview question. The development of these a priori key concepts served to organize and focus the analytic process [48]. Three different qualitative research team members, that were trained in RQA by the senior author (IML), field-tested the templates for accuracy by independently summarizing the same 5 transcripts to verify appropriateness of domains and consistency across analysts and their application of domains [49]. Summaries consisted of 2–3-page bulleted points and included exemplary quotes and an “other observations” domain for topics outside of those predetermined, thus remaining open to the development of additional domains as the analysis progressed. The analytic team met to compare and discuss summaries for similarities and differences, and to further refine and come to an agreement on template domains. Once the domains had been confirmed as identifiable and accurately accounting for the data, transcripts were divided across team members to individually summarize using the appropriate template. The senior author (IML) reviewed every other completed summary for consistency. A matrix was created for each of the different interview templates into which the bulleted summaries and quotes were transferred for systematically noting similarities and differences across participants’ responses [50]. The final step in the analytic process entailed the first (HW) (an undergraduate biology and cultural anthropology student, trained in qualitative research methods) and senior (IML) authors conducting a matrix and thematic analysis to systematically review and discuss matrices findings across transcripts to critically reflect upon and further synthesize data. This was an inductive process, in which analysts used constant comparison to compare and synthesize summaries both within and across interview type (staff or patient) to identify patterns across the data to develop themes [51].

### 2.6. Intervention Tailoring: Study Team Debriefs and Member Checking

Key findings from the group interviews with staff and patients were shared with our health education specialists within 2–3 weeks after their completion. This timeline included a one-week period for professional interview transcription. As our health educations specialists have established relationships with community partners, they serve as our implementation team, and were best suited to assist in developing practical, actionable steps to respond to concerns at individual centers. Findings and proposed action items were reviewed by the team’s senior qualitative researcher (IML) for accuracy and then discussed with the larger study team in weekly meetings to further facilitate tailoring interventions and implementation strategies to program partners’ and recipients’ needs [52]. Implementation team members subsequently engaged in member checking, meeting with program champions and other key program implementers, to share findings to collaborate on the tailoring of intervention planning and implementation and to discuss the feasibility, coordination, and timeline of any proposed changes [53]. Findings focused on responding to what staff and patients identified as the issues most critical to address to successfully treat tobacco dependence within their setting. Progress on addressing recommended action items were discussed in weekly study team meetings to ensure timely completion as well as during periodic reflections, i.e., regularly scheduled and lightly structured discussions between our implementation team members and program partners, focused on reflecting upon, guiding, and supporting implementation efforts and processes [54].

## 3. Results

The data analysis yielded four main themes that are further delineated into categories on staffs’ and patients’ perceptions, experiences, and needs regarding implementation of an evidence-based TFW program: (1) tailored materials—inclusivity and diversity, focused on adaptations to program materials to fit local contexts; (2) current practices, concerned current tobacco cessation services provided at centers and their potential for enhancement; (3) ambivalent outlooks, focused on participants’ attitudes on treating tobacco dependence that served to enable and/or hinder program implementation; and (4) additional environmental supports, focused on what staff and patients identified as necessary to support them in successfully addressing tobacco dependence.

### 3.1. Tailored Dissemination Materials

To facilitate implementation, dissemination materials were shared with staff during pre-implementation interviews to discuss their needs and gauge their feedback on current resources. These materials comprised educational posters, informational brochures, or rack cards, business-sized quit cards with the contact information for the Texas Tobacco Quitline, available in multiple languages such as English, Spanish, Farsi, and Vietnamese. Materials covered a broad range of tobacco-related topics, including smokeless tobacco, e-cigarettes, harms of smoking and smokeless tobacco, tobacco use during pregnancy, harms of tobacco use for those with mental health and substance use disorders, and several others, and represented a diverse range of individuals in terms of race, ethnicity, gender, sexual identification, and age. Program partners were asked to select a range of dissemination materials for their center, which were printed and mailed to them for free.

#### Tailored and Inclusive Dissemination Materials

The feedback from these interviews was primarily positive, as participants approved of the wide spectrum of educational resources to support tobacco cessation efforts. Staff appreciated the availability and quality of diverse and inclusive dissemination materials, and particularly appreciated that the materials were adapted to the populations that they served:

*“I think that they do target our community, our population, and I think our patients could probably relate to them”*

(Jaime, Provider, MHC #1).

In addition to appreciating the inclusive design and quality of materials, staff offered constructive feedback on how these resources could be further tailored to meet the needs of their specific patient populations. After viewing existing materials, staff were asked to share their thoughts on any changes that should be made to better suit their program, and whether they would like to see any specific populations, languages, or age groups within such resources. At multiple centers, staff expressed keen interest in receiving additional tailored materials focused on the specifics of the populations served:

Participant: *“They are good resources. I think most of the resources that are in there, we are able to use them here at the clinic.”*

(Perla, Provider, MHC #3).

Interviewer: *“Any groups of people that you think that we’ve left out that you might focus on, like the elderly … or another group?”*

Participant: *“Do you have anything more for young people?”*

(Perla, Provider, MHC #3)

Similarly, at another center that served many individuals with intellectual and developmental disabilities (IDD), there was a recognized need for materials tailored to this specific population. When asked about the usefulness of such tailored brochures, a provider indicated specific needs for IDD groups:

*“Yes…I think also a poster—a lot of the folks that I see and my practice attends, they have programs, so something that’s eye-catching, that’s heavy on the visuals I think for IDD populations.”*

(Cindy, Provider, SUTC #1).

Additionally, there was interest in educational materials on the harms of vaping in general, which was a widespread issue among teen patients:

*“The e-cigarettes are a problem…For young people that’s a very bad problem we have right now.”*

(Marta, Provider, SUTC #3).

### 3.2. Current Practices

In their pre-implementation interviews, staff were asked about their centers’ current practices related to TFW policies, tobacco-use assessments, and provision of tobacco cessation services. These practices varied across different centers, with some demonstrating comprehensive approaches while others had more limited or inconsistent practices.

#### 3.2.1. Tobacco-Free Workplace (TFW) Policies

The existing implementation of TFW policies varied across participating centers, with some having comprehensive policies while others had partial measures in place. Two of the six participating centers had established comprehensive TFW policies that disallowed tobacco use anywhere on the center premises, as confirmed by a provider:

*“No smoking on any of our property or the parking lot, whether it be the vapes or the actual cigarettes”*

(Susan, Provider, SUTC #1).

Two other program partners were considering implementing a partial TFW policy that permitted tobacco use in designated areas:

*“Right now, we haven’t incorporated anything. I think that’s why we’re where we’re at right now, why we started with you guys to educate ourselves and then start implementing…we’re not looking to be completely smoke-free,* [but having] *designated areas.”*

(Carmen, Manager, SUTC #2).

The remaining 2 centers had not adopted a center-wide TFW policy:

*“We don’t have any* [tobacco-free policy] *right now. We’ll have to implement that soon or in the future”*

(Paula, Provider, MHC #2).

Despite having comprehensive TFW policies in place, enforcement remained a challenge at some centers, as patients continued to smoke on the property:

*“*[SUTC#3] *does have a policy. We’re a tobacco-free zone. Employees are not permitted to smoke. Patients cannot smoke on the premises. Now mind you, do they smoke on the premises? Yes.”*

(Rosa, Provider, SUTC #3).

At another center, staff expressed uncertainty about the extent of their existing policy:

*“I don’t think we have any policies about that, but then I don’t think a lot of employees do smoke. I’m not too sure, honestly… I believe we just had one for no smoking inside the buildings, but I’m not sure about distance-wise or anything else other than that.”*

(Lupe, Provider, MHC #3).

This variability in TFW policies reflected the different stages of policy adoption and implementation among the centers, and the need for stronger policies in most cases.

#### 3.2.2. Tobacco-Use Assessments (TUAs)

When asked about current screening practices for tobacco use, all participating centers reported conducting TUAs during the initial intake process. At most of the centers, these were documented in the electronic health record (EHR). The comprehensiveness of these assessments varied across centers, with some asking detailed questions about different types of tobacco products, including cigarettes, e-cigarettes, vaping devices, and smokeless tobacco. As one provider shared:

*“We ask them if they smoke or not, and that could be marijuana, could be cigarettes, could be anything, really, vaping. Then we figure out how long they’ve been smoking and how heavy of a user they are, like one pack a day or half a pack.”*

(Mia, Provider, MHC #2).

Beyond the initial intake, a few centers continued to conduct TUAs at subsequent visits to monitor ongoing tobacco use:

*“It’s done pretty frequently. Usually, we try to get it with each appointment that they have”*

(Ann, Provider, SUTC #1).

Regular reassessments allowed providers to maintain an up-to-date understanding of the patient’s tobacco use habits. However, the approach to TUAs was not uniform across all centers. Staff at most centers noted their TUAs were only conducted at the initial intake, with no follow-up screenings during later visits:

*“So, the screening is usually only done in the initial assessment”*

(Marta, Provider, SUTC #3).

At one center, the lack of ongoing TUAs was also linked to their limited resources to support tobacco cessation:

*“It is a question that is asked of them, but because we don’t have the proper tools and resources to help them, the only thing we really have is the Quit Now number* [National Quitline]*. We don’t really offer like, ‘Are you wanting to quit?’ We don’t offer that until we get the resources with you all. That’s when we’ll start offering, like, ‘Hey, are you wanting to quit?’”*

(Claudia, Provider, SUTC #2).

#### 3.2.3. Current Tobacco Cessation Services

The availability and quality of tobacco cessation services differed amongst participating centers, with the majority offering limited resources and support. In many cases, the services provided were minimal, consisting primarily of educational materials and handouts. Within some centers, staff provided patients a Quitline referral; however, these were passive referrals; patients were simply given the Texas Tobacco Quitline number to contact themselves, as opposed to a direct, active referral to the Quitline (which can take multiple forms [e.g., online, fax, via EHR] and results in the Quitline calling the patient directly):

*“We do referrals, and we do education. Like the groups that we provide, we do the referrals. I mean, I think that’s the most that we provide. Like I said, I try to print some handouts, and I leave them out there on the table, but pretty much that’s all I do. We don’t have access to give them anything.”*

(Rosa, Provider, SUTC #3).

One center did offer more comprehensive services, including tobacco cessation counseling and NRT:

*“We do the tobacco cessation counseling and offer the nicotine replacement therapy, the Quitline, but to patients only as far as I know.”*

(Cindy, Provider, SUTC#1).

However, even in this case, the support was often limited in terms of available resources. One provider shared:

*“I know we provide the nicotine patches… I know from our IDD population that we service; we don’t really have much… I know that our nurses ask them and talk to them and educate them, but that’s about as far as it will go.”*

(Ann, Provider, SUTC #1).

However, even within this center, the existing systems for providing inclusive services for treating patient tobacco dependence were less than optimal. For example, delivery of tobacco cessation treatment was the responsibility of a few medical providers, rather than counselors or case-managers. Additionally, providers were not proactive in providing patients with information about tobacco cessation services; patients had to initiate these conversations with their providers to receive information about available cessation services:

*“If somebody presents with a desire to quit smoking, yes, I’ve generally referred them to medical, their doctor… I personally haven’t done anything tobacco specific as far as counseling.”*

(Jade, Provider, SUTC #1).

Notably, at one center, staff recalled having more robust services in the past, such as cessation counseling, but these had since been discontinued:

*“I remember we used to have cessation counselors, but I don’t believe we have that anymore.”*

(Perla, Provider, MHC #3).

Consistent among all centers were gaps and limitations in current tobacco cessation services provided to patients. Staff expressed interest in expanding existing and offering additional services to patients promptly, by timely provision of NRT:

*“I would say providing* [NRT] *that’s readily available at the time of the appointment… Because patients, if they had to come back, if we say, “Okay, come back in 2 or 3 days or come back tomorrow,” they won’t come back. That decision will change.”*

(Cindy, Provider, SUTC #1).

Partners were open to integrating tobacco cessation into existing services that included having patients work with peer providers who had successfully quit tobacco use:

*“Peer providers maybe, people who have quit could work with these folks so they kind of have some hope.”*

(Laura, Provider, MHC #3).

### 3.3. Ambivalent Outlooks: Attitudes Enabling or Inhibiting Addressing Tobacco Dependence

In their separate group interviews, staff and patients alike expressed ambivalent attitudes towards treating tobacco dependence. While the health benefits of tobacco cessation were recognized as undeniable by all participants, they also reported concerns and misconceptions about potentially harmful consequences of quitting tobacco for those in substance use treatment. These contradictory beliefs and attitudes thus alternately served as enablers and/or inhibitors to advancing tobacco management among patients.

#### 3.3.1. Inhibitors

Staff and patients expressed attitudes that served to hinder addressing tobacco dependence, including that patients need to smoke, or use tobacco products, to cope with the challenges of non-nicotine substance use recovery. Another shared view was that as patients have multiple challenges—substance addiction, criminal justice histories, mental health disorders, and poverty—quitting tobacco use is not a priority; these other acute issues require immediate attention, whereas tobacco dependence can be undertaken later. Staff also acknowledged the importance of timing in addressing tobacco use. As one provider stated regarding these views:

*“Because the timing is everything, because when people come in for intake assessment here, they’re just getting out of prison… They’re needing to smoke because of their anxiety and their PTSD and everything else that’s going on and trying to get everything, all their appointments situated with their parole officer here and just getting integrated out in the community. Yes, the smoking is not the priority at that time, it’s just not.”*

(Sarah, Provider, SUTC #1).

Participants from both groups also reported similar views regarding the relationship between tobacco and non-nicotine substance use. Among most staff this was expressed as a common misconception that treating tobacco and substance use concurrently would jeopardize recovery, resulting in non-nicotine substance use relapse. Some patients expressed this view as a fear that if they stopped smoking, they would relapse to their prior substance use; smoking served as a substitute for other, non-nicotine substance use:

*“I feel like if they stop cigarettes, a lot of people will use it as an excuse to relapse even… I mean personally, me stopping smoking marijuana, I started smoking cigarettes because I didn’t have marijuana. So, like I picked up the habit. I mean another addiction, but it’s my choice. If anybody else would stop smoking nicotine, they would immediately go back to their drug of choice.”*

(Juan, Patient, SUTC #2).

Yet, paradoxically, patients also perceived tobacco and non-nicotine substance use as inextricably connected, realizing that their tobacco use could drive substance use. As such, recovery from non-nicotine substance use would necessitate tobacco cessation:

*“Like in addiction because if I’m feeding that addiction, then it leads me to feed another addiction… you have to kill all the addictions because addiction isn’t a substance. It’s a state of being, right? So, for me, as long as I’m lighting a cigarette, there’s a slight chance that I’m going to light a joint. If I smoke a joint, there’s a chance that I’m going to do some crack. So, it’s all or nothing.”*

(Ruby, Patient, SUTC #1).

Some providers reported initial concerns that the TFW program might entail adopting a coercive approach that essentially shamed, judged, or pressured patients to quit, which was not compatible with their philosophy of working compassionately with patients by meeting them where they are. However, these initial apprehensions about the TTTF program were alleviated upon familiarizing themselves with the team and program:

*“I haven’t seen that coercive or the hounding* [that] *was my initial concern. Maybe not so much the coercion but just kind of—you know the mom nagging effect of every time someone sees a particular individual, they ask and it can almost—it is done out of love, but it may not be understood as that at the time and with the individuals that I work with, that was my concern before going in… I haven’t seen anything like that. It seems very respectful.”*

(Jade, Provider, SUTC #1).

However, staff and patients’ views on patients’ interest in quitting tobacco use were divergent. While staff reported patient lack of interest in quitting as one of the main inhibitors to successfully addressing tobacco dependence within their organization, others recognized that patients wanted to quit but felt frustrated and unable to do so:

*“It’s really hard to quit…most of these folks, it’s not a matter of like wanting to quit because they know it’s unhealthy. They do want to quit but they don’t feel like they can, or they tried before and failed so many times.”*

(Laura, Provider, MHC #3).

Almost all patients stated they were currently making or had made quit attempts in the past and were interested in quitting and learning more about the harms of tobacco use for themselves and their families; views that facilitate cessation efforts:

*“That’s very good information* [tobacco use is associated with at least 17 different cancers], *hearing that and now we’re aware of this. Maybe we can pass it on to our families… you got to understand too, there is help. You can be saved if you can’t stop smoking, because you don’t have to do it all by yourself.”*

(Pedro, Patient, SUTC #3).

An additional challenge to program implementation often cited by staff was lack of time to intervene upon patient tobacco dependence, given already high caseloads, limited staff and resources, and the many needs of patients:

*“We have a lot of heavy smokers. It’s* [tobacco cessation intervention] *definitely necessary in our area. It can be complicated by the fact that we’re the only provider in our rural area, which means we’re very busy, time is an issue.”*

(Carla, Provider, MHC #2)

#### 3.3.2. Enablers

Notwithstanding their aforementioned espousal of the view that concurrent tobacco and substance use treatment could jeopardize substance recovery and result in relapse, some staff reported that tobacco cessation aligned with substance use treatment. Specifically, providers reported that tobacco cessation interventions could be integrated into current addiction-focused services, which would also save time:

*“I think it* [tobacco-cessation intervention] *fits good with our work practices…We all don’t have very much time, so it’s just where we plug it, and that’s why it fits with our program because we can do it during group therapy, but we just got to make sure that the documentation, or the reports, or whatever is required of us is not overwhelming us.”*

(Rosa, Provider, SUTC #3).

Staff also recognized that tobacco dependence is a serious addiction requiring treatment like other non-nicotine substance use disorders, whose management supports a whole health approach:

*“I think that it’s important when we do get clean and sober that we don’t discount smoking, because a lot of us do, but in the long run, it’s still a form of addiction that’s killing us. So, we want to be set free from those addictions, so I totally agree with it, and I think it will help because we want our people to be clean, sober, and healthy. So, that includes smoking.”*

(Carmen, Manager, SUTC #2).

While participants in both groups supported implementation of comprehensive TFW policies as supporting and facilitating tobacco cessation efforts for staff and patients at participating centers, they also had concerns that centers would lose patients if they became completely tobacco-free:

*“It* [TFW policy] *would be beneficial for every single person here. That’s like a real big plus, but everybody’s going to cry about it…* [SUTC #2] *would lose a lot of clientele and a lot of people here would be against it. But all-in-all, I do think that it should be a non-smoking facility. That would help a lot of people that does smoke to stop smoking.”*

(José, Patient, SUTC #2).

Apprehension that adoption of a center-wide total TFW policy would cause patients to seek services elsewhere led some program partners to be reluctant about implementing a comprehensive policy—i.e., disallowing tobacco-use anywhere on center grounds. Despite these concerns, partners were amenable to initially instituting a partial TFW policy that allowed for a designated smoking area, to assess patients’ reactions to going tobacco-free and as a step to potentially acclimating them to becoming completely tobacco-free:

*“Our clientele, a lot would leave if our program was fully non-smoking…I’m a person in long-term recovery also—meaning I’ve been through a substance use program and I honestly wouldn’t—I refused to go to some because they did not have smoking allowed. So, what we’re trying to do is we’re trying to educate and get to that point…we’re not saying and guaranteeing that that’s a point…We’re willing to come in, see what we can do for our patients, what we can do for employees, and see if we can get to that goal, but if that’s not feasible, then we will probably do what we have to do to maintain our business.”*

(Maria, Manager, SUTC #3)

### 3.4. Additional Environmental Supports

By far, providers reported that additional training in treating tobacco dependence was what they needed most to support their patients and staff in gaining the confidence to successfully treat tobacco use.

*“I think just not having the adequate tools to be able to give that group [tobacco cessation counseling] to the clients would also be a factor. Rosa, she’s the one that did that intensive training with you all, so she’s learned a lot from it, but we still have other counselors that haven’t had that training. So, Rosa is super equipped to do that training with the clients… she would be okay to train our counselors to give the smoking cessation group…* [We’ve been] *unsuccessful until we get this training, to be honest with you. We have done nothing, and I think this is just our steps forward and we’re going to get there.”*

(Maria, Manager, SUTC #3).

Staff were asked to identify duration and mode of delivery of the tobacco education training delivered by TTTF to centers, as well as any additional training needs, preferences, and special topics of interest not covered in depth in our programmatic training. Providers requested training on how they could educate patients on the harms of tobacco use and finding alternative healthy coping skills, seen as an essential feature of a successful quit program:

*“Have the means to give that cessation therapy, provide alternate means of coping because I know the mental health population, a lot of them do use smoking to cope and kind of combat the negative thoughts and still their bad behaviors, so we need to find ways to kind of reinforce health—because I have that behavior* [smoking] *but that is unhealthy.”*

(Sarah, Provider, SUTC #1).

For patients, healthy alternatives to tobacco use included engaging in physical activities, working out, going outside to the park, etc., to keep them from getting bored and to distract them from their cravings for tobacco:

*“Letting us work out, like staying active, that’s what helped. I’m bored then I’ll smoke… Maybe like some days out to like the park or just having some fun to get our mind off things. That would really help because we’re literally here and we get to leave every Sunday… But maybe like an actual day where we can go have some fun without using and bonding with each other. That would actually be also beneficial because all we do is smoke. We don’t have anything to do.”*

(José, Patient, SUTC #2).

Staff and patients alike also reported providing patients with educational materials, e.g., handouts and brochures, on the harms of tobacco use, the benefits of quitting, and available resources, would be of great value to assist them in quitting:

*“Like the pamphlets they gave us that list all the chemicals that are in there. That scared us away from smoking and the types of side effects of it. I didn’t know there’s something called popcorn lung… That’s very good.”*

(Pedro, Patient, SUTC #3).

When asked what would be most helpful in assisting their patients to quit, some providers requested short videos to educate them on the harms of tobacco use:

*“Maybe like a short presentation on the dangers of smoking just to help them understand how harmful it is.”*

(Lucy, Provider, MHC #2).

Participants from both groups perceived group tobacco cessation counseling as a valuable practice to assist patients in quitting. More than half of the participating centers were planning on instituting patient group tobacco cessation counseling:

*“That’s why it* [tobacco cessation] *fits with our program because we can do it during group therapy… Because teaching it and such and having it in groups, that seems like it would be really helpful, but almost even fun to help advocate the clients about it.”*

(Carmen, Provider, SUTC #2).

When asked what they needed most to assist them in quitting, patients asked for group tobacco cessation counseling and recognized value in peer experiences:

*“Having a community or a little group that gets together and meets I think would be amazing because you’re not just talking to the nurse practitioners. You’re talking to somebody else that also has this problem and we just kind of feed off of each other… we’re talking about things that are going bad, why I want a cigarette this day. It could grow to be something more, help everybody continue…* [You can] *vent on somebody else that knows where you’re coming from, maybe has some similar, maybe not so similar, problems going on in their life. The value of one addict to another is incomparable.”*

(Amy, Patient, SUTC #1).

Provision of NRT at no cost to patients was also reported by both groups of participants as vital to helping patients and staff in managing their tobacco dependence. Some patients had prior experience of the effectiveness of using NRT:

*“The gum helped a lot, actually….The first 3 days that I was there, I was like I really want to go outside and smoke a cigarette. Then by day 6 or 7, the gum was actually helping to keep away any thought of wanting to go out. Of course, you’re at an inpatient treatment at a mental institute.* [Laughter] *You want to go out and have a cigarette, but it wasn’t the forefront of my mind anymore… That would be awesome. It would save a lot of money.”*

(Beth, Patient, SUTC #1).

Providers were grateful that they would be supplied NRT and had prior experience of its effectiveness in assisting their patients in their quit attempts:

*“We’ve had a lot of failed attempts to quit smoking on behalf of our patients. We’ve had some successful cessation—I think if we have more tools available to us, as in having the NRT here, it will help our success rate.”*

(Carol, Provider, MHC #2).

Table 2 describes how RQA findings informed actionable steps taken by the implementation team working in collaboration with our program partners to tailor interventions in response to their needs.

## 4. Discussion

This study’s findings show that while participating centers shared some common characteristics, they also had unique features and challenges which necessitated fitting the intervention to their individual settings. Use of RQA not only facilitated the adaptation of TTTF components to local contexts, but allowed for timely intervention that served to better align program development and implementation efforts between participating partners and academic researchers, fostering engagement and buy-in for program implementation. Our analysis produced targeted, actionable findings on facilitating and adapting the intervention through tailoring of program materials, enhancing current tobacco cessation practices, recognizing and addressing ambivalent attitudes that enabled and hindered program implementation, and identifying environmental supports to facilitate and sustain staff and patients in their efforts to address tobacco dependence that are critical for cancer prevention. These findings align closely with current literature highlighting the advantages of rapid qualitative methods in generating contextually relevant, real-time insights critical for intervention adaptation and improved program uptake and outcomes [31,32,56].

### 4.1. Value of Tailored and Inclusive Dissemination Materials

Staff highlighted the importance of materials being diverse, and culturally and contextually relevant to their patient populations, emphasizing that tailored dissemination materials were essential for effective communication and increased patient engagement. Additionally, tailored materials requested by staff included educational resources available in Spanish, materials for IDD populations, emphasizing the dangers of youth vaping, secondhand and thirdhand smoke, and on alternative coping skills to assist patients during their process of quitting tobacco use. All of these requested materials were developed by the study team, vetted by our program partners in a collaborative process, and printed and delivered to their centers for dissemination to facilitate patient engagement and program uptake. These findings align with prior research underscoring that diverse and culturally tailored health promotion materials significantly enhance patient receptivity and behavior change outcomes, particularly within marginalized and underserved populations [12,57,58,59,60,61].

Staffs’ request for tailored materials specific to the IDD population highlights the nuanced needs of underserved groups that often lack appropriately adapted educational resources. Existing literature suggests that health promotion and cessation efforts targeting the IDD population require specially designed materials emphasizing visual and simplified content, as traditional materials may not effectively communicate health risks or cessation strategies to individuals with varying cognitive and developmental abilities [61]. Effective interventions entail content that not only educates but also respects and addresses the specific cognitive and emotional capabilities of individuals within this population [62]. Similarly, addressing youth-oriented educational resources regarding vaping is particularly timely, given the rising popularity and perceived harmlessness of these products among younger populations [63]. Effective youth-focused interventions necessitate age-appropriate, relatable content and visuals that resonate with younger audiences and clearly communicate risks without alienating or stigmatizing young users [64,65].

Furthermore, participants identified a need for additional educational resources; ostensibly these materials were for patients, but also served to educate providers, given the historically high tobacco use rates among staff within these settings [66]. These materials we developed for patients were to motivate them to quit for themselves and their families that outlined the benefits of quitting tobacco and strategies for relapse, and ultimately, cancer prevention. This finding highlights the importance of addressing not only the physical health consequences of tobacco use but also the substantial psychological and emotional impacts associated with cessation [67]. Evidence from the research literature supports the effectiveness of media interventions in enhancing emotional engagement and facilitating behavior change, particularly when addressing complex issues like addiction and relapse prevention [67,68]. To address this concern, program partners were sent short videos (~2–3 min) available on our website (https://takingtexastobaccofree.com), that were developed by the TTTF team in English and Spanish, on the benefits of quitting tobacco use, common myths and facts about quitting tobacco, and the symptoms of nicotine withdrawal, among others.

### 4.2. Current Tobacco Cessation Practices and Policies

In this study, the evaluation of current tobacco cessation practices at participating centers revealed significant variability, further highlighting the need for comprehensive and consistently applied tobacco control policies and cessation interventions. Only two centers had implemented comprehensive TFW policies before implementing TTTF, while others either adopted partial policies or lacked formal policies altogether. The identified enforcement difficulties underscore a gap between policy adoption and practical implementation, a barrier widely recognized in existing literature [69,70]. TTTF program adaptation included working with program partners on the development and enforcement of comprehensive, organization-wide TFW policies covering all tobacco products, recognizing that such policies are associated with improved cessation outcomes [21,22]. Effective policy implementation necessitates consistent enforcement practices [21]. Through ongoing consultation with our implementation team, two of the centers that did not currently have a TFW policy in place signed on to develop and adopt a comprehensive policy. While research literature supports the greater effectiveness of a total ban on tobacco use, two centers (MHC#1, SUTC#3) expressed an interest in adopting a partial TFW policy, with the intention of allowing patients to gradually adjust to moving to a total tobacco-free environment. As an introductory step towards transitioning into a 100% tobacco-free center, instituting at least a partial ban has been proven by other studies to be effective in increasing quit attempts among patients through making tobacco-free environments more acceptability [71,72,73].

Similar variability was also noted in the frequency and consistency of TUAs amongst participating centers. Although all centers conducted initial TUAs, regular follow-up assessments were inconsistently practiced. TTTF program delivery supported centers by encouraging integration of comprehensive TUAs into every clinical encounter, aligning with evidence-based guidelines that recommend ongoing assessments for successful tobacco cessation [25]. Routine assessments not only help track patient progress but also reinforce cessation goals and quickly address relapse triggers. Implementing regular follow-up assessments alongside program delivery strengthens ongoing engagement and supports sustained cessation efforts [26]. Our participants cited logistical constraints such as limited staffing, high caseloads, and inadequate training as substantial barriers, highlighting the need for targeted training and increased organizational support noted in other studies [70,74].

The limited provision and quality of existing tobacco cessation services also represented a critical gap in this study. Services typically consisted of passive referrals to the Quitline or distribution of basic educational materials, with a lack of more proactive, evidence-based cessation methods. The TTTF program addressed these gaps by providing specialized staff training on assessing and effectively treating tobacco dependence and facilitating the integration of evidence-based cessation services, tobacco cessation counseling, and group counseling, into routine clinical practices as per clinical guidelines [23]. These included brief evidence-based practice interventions such as the 5A’s (Ask, Advise, Assess, Assist, and Arrange), a recommended, evidence-based tobacco cessation intervention that can take less than 3 min to deliver, facilitating the integration of tobacco cessation interventions with existing patient services [75]. Staff were also trained on the 5R’s (Relevance, Risks, Rewards, Roadblock, and Repetition), which is a motivational intervention used to increase patient desire to quit tobacco use [25]. Additionally, to facilitate providers in the delivery of the 5A’s, small badge cards were developed for providers listing the 5A’s and 5R’s and how to use them. Use of RQA allowed us to educate program partners on requested topics of interest that were potentially outside the scope of our training curriculum, yet relevant to the partners and warranted attention during implementation (e.g., youth vaping and treating tobacco use among pregnant patients).

Implementation team members assisted program partners on integrating active Quitline referrals into routine workflows. Additionally, emphasis was placed on expanding these services given the effectiveness of combined approaches, including counseling paired with NRT and peer counseling, which the literature consistently identifies as more effective in supporting successful cessation [18,76,77]. We also provided literature on the efficacy of combination approaches to staff, reinforcing their recognized importance and potential impact [18,25,78]. Addressing centers’ limitations through additional staff training, providing guidance on integrating active Quitline referral protocols using Quitline fax, app, and online referral portal, provision of NRT, and group tobacco cessation counseling is essential for improving cessation outcomes, and increasing knowledge of tobacco-related cancer risks. Other research supports this finding as being particularly critical among the underserved populations these centers serve [70]. A recent umbrella review compared the long-term effectiveness (≥6 months) of and adherence to pharmacological (e.g., NRT, bupropion, or varenicline) vs. non-pharmacological technology-supported (e.g., mobile phone app, text-message, or internet-based interventions like quitlines) smoking cessation interventions [79]. At 6 months, both interventions supported similar abstinence results, with the non-pharmacological technology-supported interventions showing higher adherence. At 12 months, continuous abstinence rates were similar for both interventions, despite lower adherence in the non-pharmacological group over time. These recent review findings point to the effectiveness of non-pharmacological technology-supported interventions, which due to their lower costs, may be particularly relevant for groups with limited access to care due to geographic and/or structural barriers.

### 4.3. Ambivalent Outlooks: Attitudinal Factors Impacting Tobacco Dependence Treatment

Staff and patients shared attitudes that inhibited treating tobacco dependence included endorsing tobacco use for stress management; patients have multiple, contending challenges that take precedence over tobacco cessation efforts; misconceptions that concurrent treatment of tobacco and co-occurring substance use would jeopardize non-nicotine SUD recovery; adopting a total TFW policy would result in census loss, as patients would seek services elsewhere; and patients are not interested in quitting. Yet, participants from both groups also expressed attitudes enabling treating tobacco use, such as simultaneously recognizing and supporting implementation of comprehensive TFW policies as facilitating and empowering tobacco cessation efforts among staff and patients at their centers. Staffs’ perception that tobacco cessation interventions were compatible with existing workflows as well as the seriousness of tobacco dependence, facilitated treatment integration aligned with their adoption of a whole-health approach. Adoption of such an integrated approach has long been called for by researchers as a necessary and effective strategy for addressing tobacco cessation within these settings to reduce cancer-related health disparities [80]. For patients, their experience taught them that tobacco and substance use is conjoined or simultaneous, as such, effective recovery from one entails recovery from the other. Thus, despite ambivalent attitudes, responses from both groups suggest some support for concurrent treatment of tobacco and non-nicotine substance use, which evidence has proven to be more effective in bolstering patient sobriety and preventing relapse [81,82].

Staff and patient views on patients’ desire to quit, however, were largely divergent. Staff primarily reported that patients were not interested in tobacco cessation. Most patients, however, expressed a strong desire to quit tobacco use, while some were ambivalent about quitting—i.e., feeling compelled to smoke because they were addicted and wanting to quit at the same time. A recent study comparing patient and staff reports on tobacco-related services, knowledge, beliefs, and attitudes in substance use treatment settings indicated that more patients affirmed being concerned about their smoking than what staff estimated, which may subvert the delivery of evidence-based tobacco cessation care [83]. Research on desire to quit tobacco use among those seeking services for substance use disorders consistently shows significant interest in quitting (44–80%) [84,85]. While substantial research has also disproven concerns that implementation of a total TFW policy would lead to census loss, this fear persisted among our program partners and other healthcare centers considering going tobacco-free [82,86].

### 4.4. Need for Additional Environmental Supports

Staff and patients alike were concerned with the health effects of tobacco use, as both requested healthy alternatives or substitutes, with staff requesting assistance with teaching patients healthy alternative coping skills to using tobacco products. Patients complained of being confined in centers for most of their program without any options for physical exercise and nothing to do or to distract themselves, which led to increased tobacco use out of boredom. Our findings support substantial research reports that boredom is a significant barrier to quitting smoking among priority groups, chiefly among those from under-resourced populations and those with mental health and/or substance use disorders [87,88,89,90].

Staff and patients also noted that instituting regular tobacco cessation group counseling for patients, and providing them with NRT, were primary to assisting them in quitting tobacco use. As adults with SUDs, patients felt that the support provided in group counseling sessions by others who experienced similar problems and stigma for substance and tobacco use was invaluable to bolstering successful quit attempts. Additionally, provision of NRT is an effective and evidence-based intervention, increasing cessation by 50–60% with or without additional counseling [91]. To build and reinforce an organizational culture supportive of tobacco-free living, the TTTF team recommended that program partners extend cost-free NRT to staff as well as patients.

The main barriers to program implementation noted in this study were primarily on the provider and organizational levels. As noted by Knudsen (2016), although adoption of a tobacco cessation program is an organizational decision, implementation requires that individual providers actively participate by delivering interventions to patients [92]. Prior survey studies of providers have persistently shown low adherence to tobacco cessation guidelines [93,94]. Our finding that providers largely did not proactively offer tobacco cessation services to patients, mostly due to misconceptions about concurrent treatment, has been supported by other researchers [95]. A review of barriers to smoking cessation among priority groups, including those with mental health and substance use disorders, found that lack of support to quit from healthcare providers and access to quit resources were considered main barriers on the social and community levels [96]. Unfortunately, prior research indicates that some healthcare providers have actively discouraged patient quit attempts, in favor of continuing smoking over concerns that quitting would aggravate mental health issues [97].

While there are many reasons for lack of engagement and support to assist patients with quitting on the part of providers, principal among them is lack of time, confidence, knowledge and counseling skills on how to treat tobacco dependence [98]. In a cross-sectional survey of 18 residential SUD programs, Martinez et al. (2023) compared patients’ and staffs’ perception of tobacco-related services [83]. They reported that lack of provider knowledge and confidence on how to treat tobacco use among those with SUDs was noted by patients [83]. Approximately 34% of patients affirmed that their provider had the skills required to assist them with quitting smoking, compared to 49.4% of staff who reported possessing these skills (*p* = 0.003) [83]. Furthermore, fewer patients, 16.6%, considered smoking to be a personal decision that does not concern their provider, compared to 33.1% of providers who considered patient smoking behavior as not their concern (*p* = 0.001) [83]. Other qualitative research studies indicate that many patients view being in treatment for SUDs as the perfect opportunity to also address tobacco cessation, while others are concerned about the possibility of jeopardizing SUD recovery [99]. Our findings support prior research on the discrepancies between patient and provider perspectives on patients’ concerns about their tobacco use, and indicate a missed opportunity to assist patients with quit attempts [100,101]. Along with the considerable research on active discouragement and lack of provider support to assist patients’ cessation efforts, together these findings suggest that misconceptions regarding concurrent treatment originate with providers and are communicated to and embraced by patients. In the current study, more staff than patients expressed support for these misconceptions about concurrent treatment, whereas patients also acknowledged the mutual nature of their non-nicotine substance and tobacco use and interest in quitting both.

While implementation of tobacco cessation programs is dependent upon providers actively delivering interventions to patients, without organizational leadership and infrastructure support such change cannot occur. In the current study, findings from pre-implementation group interviews indicated that the majority of centers had not adopted a TFW policy, had not received prior training on treating tobacco dependence, and cited lack of other resources (e.g., NRT and personnel) to treat tobacco dependence—all organizational challenges. To overcome educational limitations, the implementation team offered program partners additional trainings in Motivational Interviewing to assist them in overcoming low patient interest in quitting and engage them in making healthy behavior changes, as well as a Train-the-Trainer program available for in-person or online training, to accommodate center needs [25].

Finally, during periodic reflections, the implementation team shared with program champions the recommendations made by staff and patients alike on what they perceived as needed adaptations or additions to current practices, to integrate them into existing tobacco cessation care. These adaptations included the above-mentioned trainings and patient educational materials, instituting routine tobacco cessation group counseling sessions, continuing staff education, i.e., research articles shared with staff on the evidence-base of program interventions, and guidance from the implementation team on setting up systems to facilitate program implementation. As reported in our presentation of the results, one center relied on physicians to conduct TUAs, who generally have limited time with patients. Implementation team members worked with this program partner to set up a system whereby counselors would conduct the TUA with patients who are then referred to a physician for NRT assessment. These program adaptations all rely on leadership support or approval to be operationalized. Use of RQA during the formative phase of implementation allowed us to facilitate and adapt interventions to enhance fit and support implementation efforts. Ensuring that programs are tailored to individual centers not only serves to enhance appropriateness, acceptability, and feasibility of tobacco cessation interventions, but ultimately supports their fidelity and penetration [102].

### 4.5. Study Limitations and Implications

As our sample was limited to MHCs and SUTCs in Texas serving adults with SUDs, our findings cannot be said to be universal or transferable to other populations. Instead, these findings provide insight into the needs and preferences of staff and patients within these specific types of settings. A key strength of this study was its use of RQA, which offered insights into the experiences and perspectives of participants, allowing for timely responses to guide contextual efforts shaping implementation of tobacco cessation interventions in rural and medically underserved healthcare centers. Given the limited research on processes influencing successful implementation of tobacco cessation interventions within these settings, this approach captured the ongoing experiences of participants and the real-world conditions under which interventions are carried out. Additionally, the inclusion of multiple stakeholder perspectives, including providers, managers, and patients, enhanced the comprehensiveness of our findings. By exploring points of agreement and disagreement among these groups, we were able to identify multiple attitudes towards tobacco cessation, shared priorities, and areas requiring targeted attention. This study’s findings contribute valuable insights, applicable methods, and actionable findings that can be used to guide future research and intervention development in similar settings. Moreover, these findings can guide researchers in applying and adapting similar TFW programs to other settings serving the unique needs of those impacted by structural and social determinants of health. While this study focused on using RQA during the formative evaluation phase to sustainably adapt the intervention to the needs of local sites, our future work will apply these methods to summative evaluations to better assess program effectiveness outcomes.

## 5. Conclusions

This study engaged key stakeholders in TFW program development to ensure not only that the program was feasible, but also contextually appropriate and aligned with the needs of medically underserved healthcare settings. Using RQA as part of a formative research process facilitated timely identification of key enablers and barriers to program uptake, including stigma around tobacco use, limited provider training in cessation counseling, and workflow constraints that hindered consistent delivery. This allowed the researchers to have greater flexibility in collaborating with program partners to implement needed modifications in interventions targeting prevention of tobacco-related cancers [103]. By tailoring implementation to the unique needs of healthcare centers serving individuals with SUDs in medically underserved areas, who face limited access to cessation resources, this approach contributes directly to addressing tobacco-related cancer disparities in these populations. RQA was an effective method that produced targeted, actionable findings that were applied to align program content (e.g., program educational materials and trainings); delivery methods (e.g., instituting tobacco cessation group counseling, including additional providers in intervention delivery systems); and implementation strategies (such as engaging patients as active program participants/collaborators and integrating evidence-based tobacco cessation care into existing services) to center workflows, practices, and needs to enhance implementation. This study’s findings can be applied to guide the identification of site-specific areas for targeted tailoring and development of similar interventions that seek to reduce disparities in tobacco-related cancer morbidity and mortality among priority groups. These findings will be applied to a future mixed methods summative evaluation study focused on how these intervention adaptations may have impacted the effectiveness of this TFW program.

## Figures and Tables

**Table 1:** Characteristics of Participating Centers †.

Center	Average % Patients w/ BHD Mean (SD)	Enrolled Clinics *	Clinical FT & PT Staff	Total Annual Unique Patients	Total Annual Contacts	Counties Served (% Rural/MUA/P) ^
MHC#1	5.8% (5.17)	1	81	12,139	25,000	1 (100%)
SUTC#1	20.4% (31.53)	3	135	2681	90,244	7 (100%)
MHC#2	18.6% (0.89)	1	10	1000	7000	6 (100%)
MHC#3	27.2% (8.35)	3	174	10,872	40,000	2 (100%)
SUTC#2	28.25% (29.17)	2	5	40	175	1 (100%)
SUTC#3	66% (17.1)	2	77	1500	3000	1 (100%)

Note: MHC = Medical Health Center; SUTC = substance use treatment center; BHD = behavioral health disorders; FT = full-time staff; PT = part-time staff; MUA/P = medically underserved area/population. † Data are from the Leadership pre-implementation survey. * Enrolled clinics were the number of clinics overseen by a given center that chose to participate in the program. ^ = Counties were classified using Health Resources and Services Administration data and definitions as rural, containing medically underserved areas, and/or serving medically underserved populations; a value of 100% indicates all counties served met at least one of these criteria [40,41,42,43].

**Table 2:** Applying rapid qualitative analysis findings to tailor interventions.

Domain/Related Themes	Intervention Tailoring
Educational materials—staff and patients (Tailored Materials) (Ambivalent Attitudes) (Environmental Supports)	Developed educational program materials for specific groups identified by program partners (e.g., IDD populations, vaping among youth, resources in Spanish, harms of 2nd and 3rd hand smoke, and alternative coping skills) Provided videos on benefits of quitting and relapse prevention strategies (videos were created during a previous project) Providers were sent research articles to address specific concerns about treating tobacco dependence and implementing tobacco-free programs (e.g., adopting TFW policy does not adversely impact patient census) [55]
Tobacco-free workplace (TFW) policies	Hands-on guidance from implementation team to develop TFW policies aligned with center organizational values
Tobacco-use assessments (TUAs)	Guided program partners in integrating brief TUAs into regular practice and documenting them in their EHR
Tobacco cessation services (Current Practices)	Coached partners on how to integrate different tobacco care services into regular practice including Brief evidence-based tobacco cessation interventions (e.g., 5A’s, 5R’s) were added to address lack of time and high caseloads reported in these MUA/P settings Facilitating direct referral to the Texas Quitline Instituting tobacco cessation group counseling for patients Incorporating cessation services into existing addiction-focused care Expanding provision of NRT to staff as well as patients Adjusting tobacco care systems to facilitate delivery of services (e.g., including counseling staff in delivery system of cessation services)
Tobacco education training (Ambivalent Attitudes) (Environmental Supports)	Staff received specialized training on the harms of tobacco use and its treatment that incorporated specific topics requested by partners Offered the opportunity to receive additional trainings in Motivational Interviewing and a Train-the-Trainer program Program champion received a 5-day specialized Treating Tobacco Specialist training; the Motivational Interviewing and Train-the-Trainer trainings supported developing in-house expertise in delivering tobacco education training to build, support, and sustain local knowledge in treating tobacco use center-wide
Improving tobacco cessation efforts (Environmental Supports)	Staff and patients related what they considered to be essential in assisting patients to quit tobacco use (e.g., regular tobacco cessation group counseling and free NRT supply) that was communicated to program champions and leadership to encourage initiation of these measures Patients requested access to gym facilities and outings to parks and recreational areas to distract them, alleviate boredom, and offer healthy alternatives to tobacco use—requests were communicated to partners Researching and sharing with program partners a resource list of funding opportunities to support sustainment of NRT supply available within their geographic region

Note: IDD = intellectual and developmental disabilities; TFW = tobacco-free workplace; EHR = electronic health record; MUA/P medically underserved areas/populations; NRT = nicotine replacement therapy.

## Data Availability

Data are not publicly available due to privacy restrictions. The data that support the findings of this study are available from the senior author, I.M.L., upon reasonable request.
